# High intensity interval training attenuates osteoarthritis-associated hyperalgesia in rats

**DOI:** 10.1186/s12576-023-00866-4

**Published:** 2023-04-28

**Authors:** Xinwei Wang, Jiulong Song, Peng Xia, Qiang Lin, Anliang Chen, Kai Cheng, Fane Kong, Yi Shi, Xueping Li

**Affiliations:** Department of Rehabilitation Medicine, Nanjing First Hospital, Nanjing Medical University, Nanjing, 210006 Jiangsu China

**Keywords:** High intensity interval training, Hyperalgesia, Osteoarthritis, Pain

## Abstract

High-intensity interval training (HIIT) is a physical therapy that may benefit patients with osteoarthritis (OA). Cacna2d1 is a calcium channel subunit protein that plays an important role in the activity of nerve cells. However, there is currently no evidence on HIIT relieving OA-associate hyperalgesia by decreased Cacna2d1. Our study established the OA rat models with intra-articular injection of monosodium iodoacetate (MIA). This experiment was divided into two stages. The first stage comprised three groups: the control, OA, and OA-HIIT groups. The second stage comprised two groups, including the AAV-C and AAV-shRNA-Cacna2d1 groups. OA rats were positioned at the L5–L6 segments, and 20 µl of AAV virus was injected intrathecally. The pain threshold, cartilage analysis, Cacna2d1, and pain neurotransmitters were measured and compared. The pain threshold was significantly lower in OA rats than in control rats from the first to the tenth week. Starting from the sixth week, OA-HIIT rats exhibited significantly increased pain thresholds. The expression of Cacna2d1 increased in OA rats. Moreover, the knockdown of Cacna2d1 significantly down-regulated the expression of c-Fos, SP, and Vglut2 in the posterior horn of the spinal cord. In conclusion, HIIT attenuates OA-associated hyperalgesia, which may be related to the down-regulation of Cacna2d1.

## Introduction

The prevalence of osteoarthritis (OA) is increasing with the increase in aging and obese people over time. Currently, the global incidence of OA has exceeded 7% of all the populations, of whom 15–40% suffer from chronic pain and are resistant to therapeutic drugs [1]. Hyperalgesia is a group of symptoms that manifest severe pain caused by mild stimulation, followed by insomnia, fatigue, anxiety, and depression, consequently affecting the quality of life of patients with OA [2, 3]. Hyperalgesia is mainly driven by inflammatory responses, and abnormal neuronal excitability. As a result, even conventional or subthreshold afferent stimuli can cause pain [4]. Peripheral and central sensitization are the two primary forms of hyperalgesia. Peripheral sensitization refers to the hypersensitization of primary neurons and their terminals by noxious stimuli. In contrast, central sensitization refers to the thalamus and cerebral cortex neuronal excitability [5]. The molecular targets of pain sensitization caused by OA are not completely clear. Identification of its specific molecular targets is the key to alleviate OA-associated pain.

The N-type calcium channels are potential therapeutic targets for regulating inflammation and pain [6]; however, N-type calcium channels have multiple isoforms of proteins, which are predominantly expressed in the dorsal root ganglia of the spinal cord [7]. Cacna2d1, an important isoform of the N-type calcium channel proteins, may be involved in the occurrence of OA-associated hyperalgesia [8]. Cacna2d1 contributes to the development of hyperalgesia; however, the knockdown of Cacna2d1 may alleviate pain [9, 10]. Some studies have verified that Cacna2d1 promotes the occurrence and development of secondary hyperalgesia, and that blockade of Cacna2d1 can significantly relieve pain [11]. In terms of the fact that long-term use of opioid receptor drugs (such as morphine) can cause drug resistance and hyperalgesia [12], Cacna2d1-deficient mice exhibited attenuated pain sensitization, with analgesic effect preserved, when administered with long-term morphine intraperitoneally [13].

High-intensity interval training (HIIT) is a physical therapy, characterized by a short-time high-intensity exercise with an interval period of resting recovery. Compared with conventional exercise, HIIT can improve the exercise efficiency quickly, strengthening lower limb muscle strength and relieving pain, and increasing the cardiopulmonary metabolism of OA patients [14-19]. Studies have shown that Tai Chi, Baduanjin, cycling, and other exercises can reduce local inflammatory cytokines in the OA joint and the inflammatory infiltration of pain neuron fibers, block the pain-conducting pathway, and consequently relieve OA pain [20]. Different exercise may share a common pain pathway in knee OA patients. Exercise interventions regulate brain areas involved in the dopaminergic neurotransmitter systems to achieve analgesia [21]. In addition, exercise training can reduce hyperalgesia by regulating the ion channels of spinal neurons, affecting the release of pain-causing substances, and inhibiting the activation of glial cells in the posterior horn of the spinal cord [22]. However, whether HIIT can alleviate OA hyperalgesia is questionable, and more research is needed to explore its specific mechanism.

## Materials and methods

### Ethics statement

We have included an ARRIVE checklist to show that we have conformed to the ARRIVE guidelines. All animal experiments were performed following the Chinese Guidelines of Animal Care and Welfare, and the present study was approved by the Animal Care and Use Committee of China.

### Rat model with OA

Female 8-week-old Wistar rats (weighed 200 ± 20 g; *N* = 30) were housed under a light to dark cycle alternated every 12 h with temperature and humidity (24 ± 2  ℃ and 50 ± 5%) maintained, and they had free access to food and tap water at all times. All animals were housed in group of 3 per cage (cage type: 46 cm × 35 cm × 20 cm ventilated cage). After 1 week of adaptive feeding, rats were anesthetized by intraperitoneal injection of 10% chloral hydrate (0.33 ml/100 g). OA model was induced in the right knee by a single intra-articular injection of monosodium iodoacetate (MIA) (1 mg in 50 μl of sterile saline; *N* = 24; Sigma-Aldrich, St. Louis, MO, USA) as described elsewhere [23], and control rats received an equal volume of sterile saline by intraarticular injection (*N* = 6).

### Research design

There are two stages to this experiment. In the first stage, the rats were randomly divided into three groups (*N* = 6/group), including the control, OA, and OA-HIIT group. OA and OA + HIIT rats received MIA injection, whereas control rats were injected with an equal volume of saline. The OA-HIIT rats were administered a HIIT exercise prescription. The control rats and OA rats exercised at 10 m/min speed for 0.5 h. In the second stage, we knocked down Cacna2d1 using adeno-associated virus (AAV, PT4409 rAAV-hSyn-mCherry-5'miR-30a-shRNA (Cacna2d1)-3'miR-30a-WPREs, Serotype 2/9, 5.52e+12vg/ml, Brain VTA, Wuhan, Hubei, China) to verify whether Cacna2d1 could regulate pain neurotransmitters in the posterior horn of the spinal cord. The OA rats were randomly divided into AAV-C and AAV-shRNA-Cacna2d1 groups (*N* = 6/group). The AAV-shRNA-Cacna2d1 rats were intrathecally injected 20 µl of the AAV virus, whereas AAV-C rats were intrathecally injected 20 µl of the control virus (PT2321 rAAV-hSyn-mCherry-5'miR-30a-shRNA (scramble)-3'miR-30a-WPREs, Serotype 2/9, 5.74e+12vg/ml).

### HIIT protocol

All rats underwent a familiarization period on the treadmill (YS101, Tianjin, China) at a low speed of 5 m/min-10 m/min for five consecutive days. They gradually increased the speed and intensity of exercise until all rats were used to the treadmill environment and speed change. During the familiarization, four rats were excluded due to their inability to adapt to running behavior. The number of rats was also supplemented to ensure that all rats in each group could complete the exercise. Maximum oxygen consumption (VO_2_max) was measured as described previously [24, 25]. VO_2_max baseline results for prescribing the HIIT were used as follows: 39.82 ± 2.976 ml/kg min. HIIT included low-speed running at a speed of 15 m/min for 4 min, followed by high-speed running at 25 m/min for 1 min, and cool-down at a constant speed of 10 m/min for 1 min (6 bouts of exercise/recovery interval). The exercise prescriptions were performed in 5 sessions per week and continued for 6 weeks.

### Pain threshold assessment

Mechanical withdrawal threshold (MWT) was evaluated by the von Frey testing (NC12775-99, North Coast, USA). After acclimating to the test apparatus for 20 min, rats received a gradually increased perpendicular pressure (2, 4, 6, 8, and 15 g) by the "Up & Down" method at the right plantar surface of the hind paw. The filaments should be bending to "C" or "S" shape and maintained for 6–8 s. Animals not responding were marked as negative "O" and those responding (pinching or licking) were marked as positive "X". Results of the mechanical pain threshold testing were calculated using the following formula: 50% threshold (*g*) = (10^[*X*^_f_^+ *K*^_δ_^]^)/10000, where *X*_f_ refers to value (in log units) of the final von Frey hair used, and the constant *k* refers to a coefficient value for the pattern of positive/negative responses and *δ* = 0.224 [26].

### Proteomics

After euthanizing rats by intraperitoneal injection of 10% chloral hydrate (0.5 ml/100 g), their lumbar spine was removed, and their spinal cord tissue was removed entirely. These spinal cord samples were transferred into the lysis buffer (1% SDS, 8 M urea, 1 mg/ml protease inhibitor cocktail), vortexed and lysed for 30 min on ice, and then homogenized for 2 min in ice using an ultrasonic homogenizer (Voshin, Wuxi, Jiangsu, China). The homogenate was centrifuged at 12,000 rpm for 15 min at 4 ℃, and its supernatant was collected. The protein concentrations of the supernatant were determined to use the BCA protein assay kit (Keygenbio, Nanjing, China), and then 100 μg of protein per condition was transferred into another Eppendorf tube and the final volume was adjusted to 100 μL with 8 M urea. The labeled peptide samples were re-dissolved in solvent A (0.1% formic acid in water) and analyzed by on-line nanospray LC–MS/MS on Orbitrap Fusion™ Lumos™ Tribrid™ coupled with EASY-nLC 1200 system (Thermo Fisher Scientific, MA, USA). The differentially expressed proteins between the two groups were analyzed by the Kyoto Encyclopedia of Genes and Genomes (KEGG) pathway. The enriched KEGG pathway mapping was performed on KEGG (v2.0, http://www.genome.jp/kegg/), a database resource with large-scale datasets obtained from high-throughput experimental technologies [27].

### AAV intervention

Rats are removed from their lower back hair and disinfected with 75% alcohol. The intrathecal injection site is lumbar segments 5–6 (L5–L6) intervertebral space. We used a micro-syringe to push 20 μl of AAV into the spinal canal. The rat tail trembling or sudden flicking demonstrates successful injection [28].

### Safranin O-fAST green staining

After rats were euthanized, the cartilage was harvested and fixed in 4% paraformaldehyde for 3 days. After decalcification, dehydration, and paraffin embedding, knee cartilage tissue was sectioned in a coronal plane with a thickness of 3 μm using a pathological slicing machine (HistoCore BIOCUT, Leica, Germany). Cartilage sample slices were stained with safranin-O dye (Free Thinking, Nanjing, China) for 1 h and then washed in distilled water. After being soaked for 5 s in 50%, 70%, and 80% alcohol, respectively, slides were washed with a fast green dye (Free Thinking, Nanjing, China) for 2 h. Next, sections were washed briefly with absolute ethanol for 30 s, cleared with xylene, and finally sealed in optical resin. The cartilage tissue was observed under a light microscope (Olympus BX51, Japan) and evaluated by Mankin score, including whether the cartilage surface was regular, whether the cartilage staining was absent, whether there was fissure formation (Table 1) [29].

**Table 1:** The Mankin rating scale

Index of score	Score
I cartilage structure	
Even surface	1
Uneven surface	2
Fibrillated and fissured within superficial zone only	3
Fissures and erosions extending below the surface zone, without extending beyond the radial zone	4
Fissures and erosions extending into the deeper zone	5
II chondrocyte loss	
Loss extending into superficial zone	1
Loss extending into midzone	2
Loss extending into radial zone	3
III matrix distribution	
Normal staining	1
Moderate loss in staining	2
Severe loss in staining	3
No staining	4
IV chondrocyte cloning	
No clusters	1
Chondrocyte clusters in superficial zone	2
Chondrocyte clusters in superficial to midzone (less than four cells)	3
Chondrocyte clusters of more than four cells located in superficial to midzone, or chondrocyte clusters in deeper zone	4

### Immunofluorescence

The lumbar spinal cord tissues (L4–L5) were fixed in 4% paraformaldehyde for 6 h. The dehydrated tissues were successively dipped in wax, embedded, and sectioned in transverse sections with a thickness of 3 μm. Tissue sections were immersed in xylene for 25 min, followed by hydration in 95%, 80%, and 70% absolute ethanol for 2 min. The tissue sections were boiled in repair solution at 95 °C for 15 min and naturally cooled. The 10% goat serum (Keygenbio) blocked for 20 min at room temperature. The anti-Cacna2d1 (1:250, Novus Biologicals, New York, NY, USA), anti-SP (1:100, Santa Cruz Biotechnology, Dallas, Texas, USA), anti-Vglut2 (1:500, Abcam, Cambridge, UK), and anti-c-Fos (1:100, Santa Cruz Biotechnology, Santa Cruz, USA) were incubated at 4 °C for 16 h. Sample slices were washed three times in PBS and then incubated in species-specific secondary antibodies (1:1000 Goat anti-mouse IgG or 1:400 Goat anti-rabbit IgG, Keygenbio) at room temperature for 1 h. Nucleus was then stained by mounting medium with DAPI (1:200, Histova, Beijing, China). The image was observed using a digital pathology image scanner (version 12.3.3.5048, Leica, Wetzlar, Germany) [30]. The density of positive cells in the posterior horn of the bilateral spinal cord was analyzed quantitatively using Image J software.

### Western blotting

The spinal cord tissue of the L4–L5 was lysed in RIPA buffer (Keygenbio), and protein concentrations were measured by the BCA protein assay kit (Keygenbio). Protein samples were separated on a 4–20% SDS-PAGE gel and then transferred onto polyvinylidene fluoride membranes. After being blocked with blocking buffer (NCM Biotech, Suzhou, Jiangsu, China) for 10 min at room temperature, membranes were incubated overnight at 4 °C with primary antibodies anti-SP (1:1000; Santa Cruz Biotechnology), anti-Vglut2 (1:1000; Abcam), anti-c-fos (1:100; Santa Cruz Biotechnology), or anti-Gapdh (1:1000; Bioss, Beijing, China). All primary antibodies were diluted with TBST solution. Finally, the membranes were incubated with secondary antibodies goat anti-mouse IgG (1:5000, Keygenbio) or goat anti-rabbit IgG (1:5000, Keygenbio) for 2 h. The bands were visualized using an enhanced chemiluminescence reagent (Thermo Fisher) and quantified by the Image 4000 system (Tanon 6600, Beijing, China) [31].

### Statistical analysis

Shapiro–Wilk test was used for normality evaluation. Data were presented as mean ± SD and analyzed by the SPSS 22.0 (SPSS Inc., Chicago, USA) and GraphPad Prism 9.0 (GraphPad Software Inc., San Diego, CA, USA). Independent sample *T* test was used for comparison between the two groups. One-way analysis of variance was used for statistical comparisons between the different groups. Repeated measures analysis of variance was used for pain threshold data. Tukey HSD, LSD, and Bonferroni test methods were used as post hoc tests.* P* < 0.05 was considered statistically significant.

## Results

### HITT attenuates OA-induced hyperalgesia

**Fig. 1:**
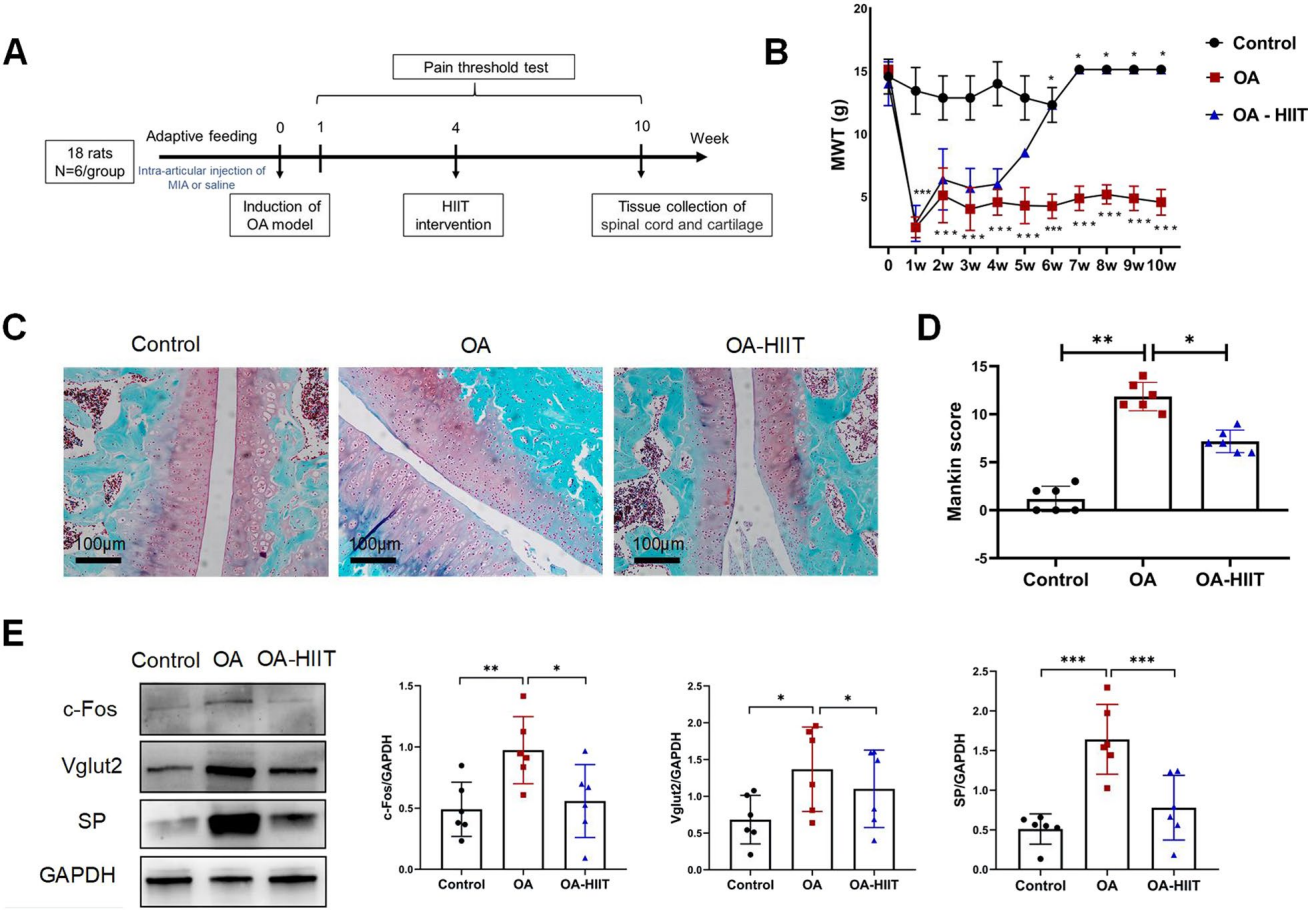
HITT attenuates OA-induced hyperalgesia. **A** Research design and timeline. Twelve rats received an MIA injection to build an OA model after 1 week of adaptive feeding and exercise. Six rats received saline injections as control group. After 4 weeks, OA-HIIT rats were subjected to the HIIT treatment for six consecutive weeks, and control rats and OA rats exercised freely on the closed treadmill. Pain thresholds were assessed weekly in all rats, and the right knee joint and spinal cord was extracted after the 10th week. **B** The von Frey testing detected rats' mechanical withdrawal threshold (MWT). *n* = 6/group, repeated measures analysis of variance. Tukey and Bonferroni tests method was used as a post hoc test. **C**,**D** Representative Safranin-O Fast green staining image (scale bar = 100 μm) and Mankin score of cartilage (n = 6 /group). Blue staining for bone and red staining for cartilage. One-way analysis of variance, the Tukey and LSD tests method was used as a post-hoc test. **E** Representative western blot image of spinal cord tissue ( L4–L5) and quantitative analysis of c-Fos, Vglut2, and SP (*n* = 6 /group). One-way analysis of variance, the Tukey and LSD tests method was used as a post hoc test. * *P* < 0.05, ** *P* < 0.01, *** *P* < 0.001 as indicated

After the fourth week of MIA injection, OA-HIIT rats began to exercise for 6 weeks. All experimental rats were subjected to the von Frey testing to measure their pain threshold (Fig. 1A). In the pain testing (Fig. 1B), the pain threshold was significantly lower in OA rats than in control rats from the 1st to 10th week (*P* < 0.001). Starting from the 6th week, OA-HIIT rats exhibited significantly increased pain thresholds compared with OA rats (*P* < 0.05). The cartilage was analyzed by safranin O fast green staining (Fig. 1C–D). The Mankin score was significantly higher in OA rats than in control rats (*P* < 0.05). Further, compared with OA rats, OA-HIIT rats displayed significantly decreased Mankin scores (*P* < 0.05). On the other hand, western blotting analyzed pain neurotransmitters in the spinal cord (Fig. 1E). OA rats exhibited significantly increased expression of c-Fos, Vglut2, and SP compared with control rats; however, use of HIIT reversed their increased expression levels in OA rats (*P* < 0.05).

### HIIT decrease Cacna2d1 expression in OA rats

**Fig. 2:**
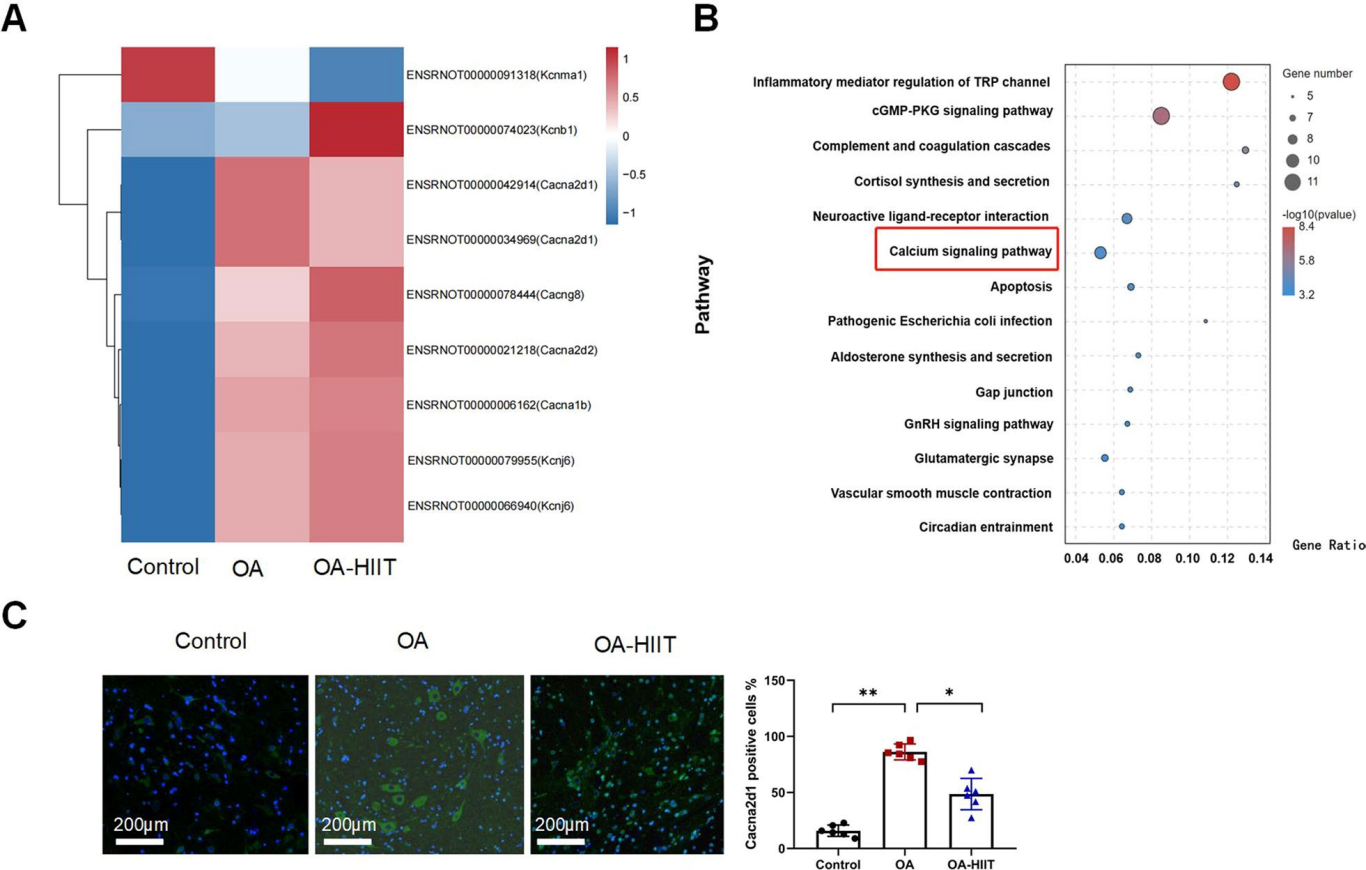
HIIT decrease Cacna2d1 expression in OA rats. **A** Heat map analysis of the differential proteins in control rats, OA rats, and OA-HIIT rats. **B** KEGG pathway enrichment analysis of differential pathways. **C** Representative images of immunofluorescence (scale bar = 200 μm) and quantitative analyses of Cacna2d1 in the posterior horn of the spinal cord. Blue is stained for the nucleus, and green are marker proteins. *n* = 6/group, one-way analysis of variance was used for comparisons between groups, the Tukey and LSD tests method was used as a post hoc test. *P < 0.05, **P < 0.01 as indicated

The proteomics approach was applied to identify protein changes in the control rats, OA rats, and OA-HIIT rats. There were fourteen differential pathways in different groups of rats. The larger the circle in the bubble plot, the more genes were enriched in the pathway. Heat map of significantly up-regulated and down-regulated proteins associated with ion channels among different groups, four of which were related to the calcium signaling pathway, including Cacna2d1, Cacna2d2, Cacna1b, and Cacng8 (Fig. 2A,B). The expression of Cacna2d1 up-regulated in OA rats. HIIT down-regulated Cacna2d1 level. To further explore the expression level of Cacna2d1 in the posterior horn of the spinal cord, we investigated Cacna2d1 in the control rats, OA rats, and OA-HIIT rats using immunofluorescence (Fig. 2C). Compared with control rats, OA rats exhibited significantly increased expression levels of Cacna2d1 (*P* < 0.05); however, HIIT markedly reversed expression levels of Cacna2d1 in OA rats (*P* < 0.05).

### Cacna2d1 is involved in regulating the release of pain neurotransmitters

**Fig. 3:**
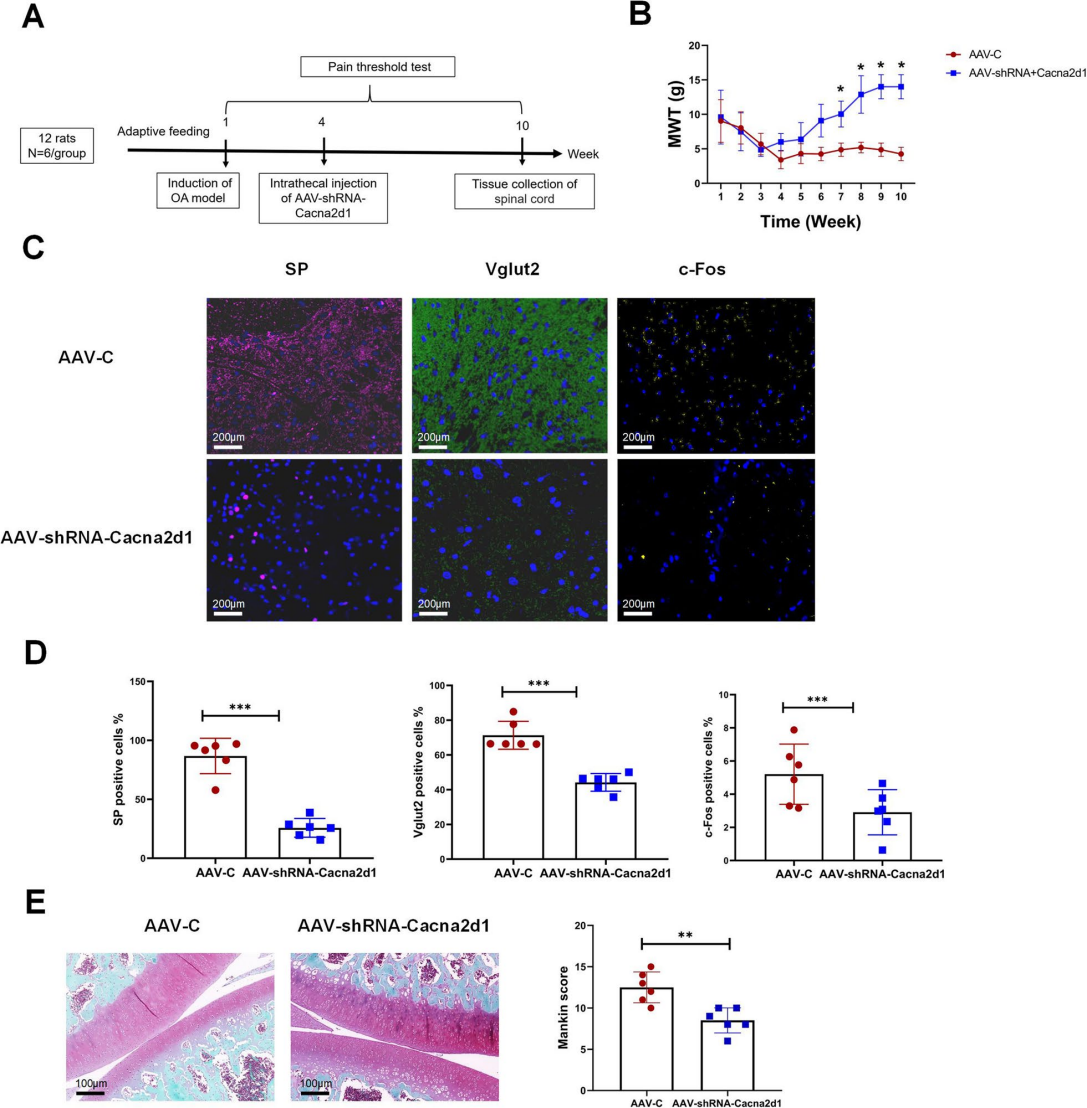
Cacna2d1 is involved in regulating the release of nociceptive neurotransmitters. **A** Experimental timeline. In the fourth week of the rat OA model, the AAV specifically knocked down Cacna2d1 by intrathecal injection. AAV-C is as control. Pain thresholds were assessed weekly. **B** AAV-shRNA- Cacna2d1 rats increased the pain thresholds from 7th week to 10th compared with AAV-C rats. *n* = 6 rats/group, repeated measures analysis of variance, Tukey and Bonferroni tests was used as a post hoc test. **C**–**D** Representative images of immunofluorescence (scale bar = 200 um) and quantification of Vglut2, SP, c-Fos in the posterior horn of the spinal cord. *n* = 6/group. The independent sample T test was used for comparison, and Levene's test for homogeneity of variance. *P < 0.05, ***P < 0.001 as indicated. **E** Representative Safranin-O staining image (scale bar = 100 μm) and Mankin score (*n* = 6/group). The independent sample T-test was used for comparison, and Levene's test for homogeneity of variance. **P < 0.01 as indicated. Blue staining for bone and red staining for cartilage tissue

To verify whether Cacna2d1 could regulate pain neurotransmitters in the posterior horn of the spinal cord, we knocked out Cacna2d1 using AAV. After adaptive feeding for 1 week, rats received MIA injection to build an OA model. OA rats were injected in the fourth week with AAV-C or AAV-shRNA-Cacna2d1. All rats were subjected to the von Frey testing (Fig. 3A). As shown in Fig. 3B, the pain threshold significantly increased in AAV- shRNA-Cacna2d1 rats at the seventh week (*P* < 0.05). We further detected pain neurotransmitters in the spinal cord by immunofluorescence (Fig. 3C,D). Compared with AAV-C, AAV-shRNA-Cacna2d1 rats exhibited significantly decreased expression levels of Vglut2, SP, and c-Fos (*P* < 0.05). The Mankin scores remarkably decreased in AAV-shRNA-Cacna2d1 rats compared with AAV-C rats (*P* < 0.05, Fig. 3E).

## Discussion

In this study, we determined the effects of HIIT on OA-induced hyperalgesia and the involvement of Cacna2d1 in such effects. We found a persistently lower pain threshold in the OA rats, accompanied by increased Cacna2d1 expression. However, HIIT reversed increased expression levels of Cacna2d1 in OA rats, and knockdown of Cacna2d1 in rats with OA inhibited the release of the pain-causing neurotransmitters.

We chose to induce the OA model by intra-articular injection of MIA. MIA can stimulate the inflammatory system in the joint and further lead to the destruction of articular cartilage, which is more effective in eliciting a pain response. Previous studies have demonstrated that the MIA-induced OA model was characterized by varying degrees of cartilage destruction and ATF-3 immunoreactivity expressed in the posterior horn of the spinal cord, which is a marker of neuronal injury and suggests the development of neuropathic pain [32]. OA model can also be induced through anterior cruciate ligament transection (ACLT) or meniscectomy, leading to the instability of the joint structure, which is not suitable for exercise in the later stage of the experiment.

**Fig. 4:**
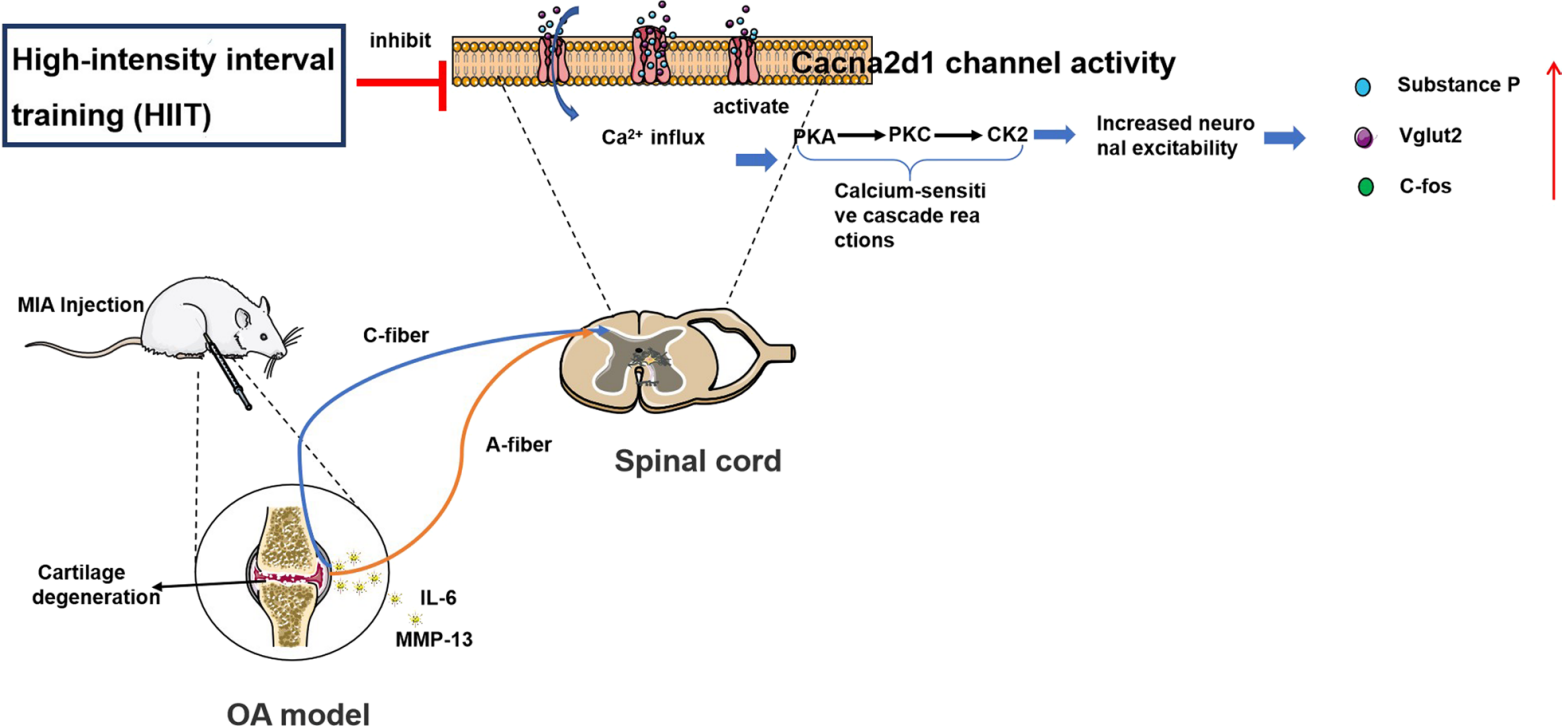
Schematic illustration showing how intra-articular inflammatory mediators are delivered to the posterior horn of the spinal cord and finally cause pain sensitization. The nociceptive stimuli signals are transmitted to the posterior horn of the spinal cord through A fibers and C fibers, leading to increased Cacna2d1 channel activity. The increased influx of Ca^2+^ further triggers a calcium-sensitive cascade, releasing pain neurotransmitters (Vglut2, SP, and c-fos). Neurons have higher excitability under the long-term infiltration of inflammatory mediators and pain neurotransmitters, which increases Ca^2+^ influx and continuously releases pain neurotransmitters forming a vicious cycle reaction, leading to hyperalgesia

The pathological manifestations of OA are cartilage degeneration and bone hyperplasia [33]. Cartilage injury activates the mononuclear macrophage system, stimulates peripheral nociceptors, and pain neurotransmitters continue infiltrating pain receptors in the central nervous system [34, 35]. The mechanism of OA leading to hyperalgesia, as shown in Fig. 4, is that intra-articular inflammatory mediators are transmitted to the posterior horn of the spinal cord through afferent fibers in OA rats, leading to increased Cacna2d1 channel activity. The increased Ca^2+^ influx further releases pain neurotransmitters. Inflammatory and pain neurotransmitters increase central neuron excitability, eventually leading to hyperalgesia. HIIT inhibit the expression level of Cacna2d1, reduces the pain neurotransmitters and inflammatory infiltration, improving OA-associated hyperalgesia.

Our results suggested that the rats had a reduced pain threshold compared with control rats after the first week of MIA injection, and the low pain threshold persisted until the tenth week. We also found the expression of SP, Vglut2, and c-Fos (all of which are pain-inducing substances) increased in the spinal cord of OA rats. These results indicate the presence of hyperalgesia in OA. The excessive influx of Ca^2+^ can cause the release of excitatory neurotransmitters and cause pain sensitization [36]. To investigate which key proteins regulates OA hyperalgesia, we used proteomics to analyze protein differences in the central nervous system. Heat map analysis and immunofluorescence showed that HIIT can down-regulate significantly increased expression of Cacna2d1 in OA rats.

Previous studies have shown that Cacna2d1 is an essential isoform protein of the N-type calcium ion channel, which involved in the regulation of the pain transmission pathway [37]. The expression level of Cacna2d1 was correlated with the degree of cartilage lesions in OA [38]. We found that the expression level of Cacna2d1 was significantly increased in OA rats. Therefore, we used AAV to specifically knock down Cacna2d1 in the spinal cord of OA rats, and found that the expression of SP, Vglut2, and c-Fos was decreased in the AAV-shRNA-Cacna2d1 rats, suggesting that Cacna2d1 can facilitate the release of pain-causing substances in the posterior horn of the spinal cord. Our results suggest that HIIT exerts beneficial effects on OA-induced pain though down-regulation of Cacna2d1. HIIT affects Cacna2d1 expression in the central nervous system and may be related to the following mechanisms. During exercise, the nervous system releases some neurotransmitters, such as norepinephrine and acetylcholine, which are related to regulating calcium channels and may affect the expression of Cacna2d1 [39]. In addition, exercise regulates some calcium signaling and affects cellular function and metabolism, decreasing Cacna2d1 expression [40].

The effects of HIIT on OA cartilage are complex and diverse, and there may be some positive and negative effects. Some studies have shown that HIIT can enhance skeletal muscle strength and improve motor function in patients with OA, enhancing the metabolic status of articular cartilage and thereby reducing hyperalgesia [14, 41]. However, there are some objections that HIIT can cause greater joint stress, which may lead to cartilage destruction and pain aggravation [42, 43]. Therefore, it is recommended that OA patients, under the guidance of professional doctors, comprehensively consider the efficacy and safety of HIIT and develop a personalized training program to reduce pain and improve health and quality of life [44].

The major limitation of this study is that the MIA-induced OA model has a non-physiological initiation and a relatively rapid onset compared to surgical models [45]. Although it is well accepted as a model for OA pain [46], its applicability to idiopathic OA and the human condition remains unclear. Moreover, this study uses weight-bearing treadmill running, which may not be feasible for OA patients with severe deformities. Therefore, in future clinical trials, we can develop HIIT exercise prescriptions on the anti-gravity treadmill or bicycles [47, 48]. Female animals were chosen for the study in terms of the fact that the incidence of OA is much higher in females than in males, which is more closely related to the epidemiology and etiology of OA.

In summary, the present study reveals that HIIT attenuates OA-associated hyperalgesia in rats, which may be related to the down-regulation of Cacna2d1. Moreover, Cacna2d1 can regulate nociceptive neurotransmitters in the posterior horn of the spinal cord. Our study provides a theoretical basis for HIIT to alleviate OA pain.

## Data Availability

All the data and materials are available.
